# Interprofessional collaboration in primary care: what effect on patient health? A systematic literature review

**DOI:** 10.1186/s12875-023-02189-0

**Published:** 2023-11-29

**Authors:** Céline Bouton, Manon Journeaux, Maud Jourdain, Morgane Angibaud, Jean-François Huon, Cédric Rat

**Affiliations:** 1 Department of General Practice, Faculty of Medicine, University of Nantes, 1, Rue Gaston Veil, 44035 Nantes, France; 2 Primary Care Federative Department, Faculty of Medicine, University of Nantes, Nantes, France; 3 Faculty of Pharmacy, University of Nantes, Nantes, France

**Keywords:** Interprofessional collaboration, Primary care, Cardiovascular, Polypathology

## Abstract

**Background:**

In a period of change in the organization of primary care, Interprofessional Collaboration (IPC) is presented as one of the solutions to health issues. Although the number of inter-professional interventions grounded in primary care increases in all developed countries, evidence on the effects of these collaborations on patient-centred outcomes is patchy. The objective of our study was to assess the effects of IPC grounded in the primary care setting on patient-centred outcomes.

**Methods:**

We conducted a systematic literature review using the PubMed, Embase, PsycINFO and CINAHL databases from 01/01/1995 to 01/03/2021, according to the PRISMA guidelines. Studies reporting the effects of IPC in primary care on patient health outcomes were included. The quality of the studies was assessed using the revised Downs and Black checklist.

**Results:**

Sixty-five articles concerning 61 interventions were analysed. A total of 43 studies were prospective and randomized. Studies were classified into 3 main categories as follows: 1) studies with patients at cardiovascular risk (28 studies)—including diabetes (18 studies) and arterial hypertension (5 studies); 2) studies including elderly and/or polypathological patients (18 studies); and 3) patients with symptoms of mental or physical disorders (15 studies). The number of included patients varied greatly (from 50 to 312,377). The proportion of studies that reported a positive effect of IPC on patient-centred outcomes was as follows: 23 out of the 28 studies including patients at cardiovascular risk, 8 out of the 18 studies of elderly or polypathological patients, and 11 out of the 12 studies of patients with mental or physical disorders.

**Conclusions:**

Evidence suggests that IPC is effective in the management of patients at cardiovascular risk. In elderly or polypathological patients and in patients with mental or physical disorders, the number of studies remains very limited, and the results are heterogeneous. Researchers should be encouraged to perform studies based on comparative designs: it would increase evidence on the positive effect and benefits of IPC on patient variables.

**Supplementary Information:**

The online version contains supplementary material available at 10.1186/s12875-023-02189-0.

## Introduction

The development of primary care, defined as a model of care that supports first-contact, accessible, continuous, comprehensive and coordinated person-focused care, is a global priority [1]. Studies have already shown that most patients are treated in a primary care setting [2, 3]. Most patients suffering from common diseases such as hypertension, diabetes, chronic obstructive pulmonary disease and asthma only consult a primary care provider [4]. The ageing of populations, the growing importance of chronic pathologies, the international shortage in the health care workforce [5], and the growing complexity of care pathways call for development of new modalities of practice in primary care.

Functioning in a primary care team, based on Interprofessional Collaboration (IPC), is widely supported by the health authorities in France [6], similar to existing models in several countries [7]. IPC is defined as several health workers from different professional backgrounds providing comprehensive services by working with patients, their families, and other caregivers [8]. This definition can be supplemented by the need for contact, negotiation and interaction among health care professionals. As this concept is recent and vast, several terms have been used, but the term IPC is the most currently used [9]. Part of these IPC teams regroup professionals in the same practice. Many practices work as a team of GP's, nurses (including Advanced Nurse Practitioners), paramedics (including Advanced Clinical Practitioners), clinical pharmacists, physiotherapists, physician associates and others. In this model, an integrated and collaborative approach to patient care has been developed. The literature on this subject shows a recent emulation with numerous articles in many journals [1, 7–11]. Several authors have reported that working as a team is a source of satisfaction among professionals [7, 10]. A 2018 literature review reported how primary care teams were formed [11], but it did not report any information on the effect of these organizations on patient-centred outcomes.

Some authors have focused on the team-based approach for specific pathologies for which collaborations largely mobilize specialists and hospital professionals, but these investigations concern secondary rather than primary care services [12–14]. While a recent literature review [15] investigated the effect of IPC in a primary care setting on adults with diabetes and/or hypertension, we wanted to further investigate which areas of treatment and primary care team organization had an effect on patient-centred outcomes.

A better understanding and better characterization the composition of IPC teams and the health fields in which this collaboration would be relevant and effective for patients is important for decision-makers and professionals who wish to engage in the evolution of their practice. Which professionals are involved in IPC? For which treatments and illnesses have results been obtained? For which treatments and diseases have we not obtained convincing results?

The objective of this study was to assess the effects of IPC grounded in primary care setting on patients-centred outcomes.

## Methods

This systematic review was conducted according to the PRISMA guidelines [16]. We searched for studies published between January 1st, 1995 and March 1st, 2021. To ensure that we found all research articles published by the various health professionals, we chose to increase the number of search engines normally used. The following databases were searched: PubMed, Embase, PsycINFO and CINAHL. Additional articles that were found by hand searching the references were also reviewed. The following research algorithms were used 1) PubMed: ("Intersectoral Collaboration"[Mesh] OR "Cooperative Behavior"[Mesh] OR "Patient Care Team"[Mesh:NoExp]) AND ("Primary Health Care"[Mesh]) AND ("Outcome and Process Assessment, Health Care"[Mesh]); and 2) Embase, PsycINFO and CINAHL: « intersectoral collaboration», « cooperative behaviour», « patient care team» AND « primary health care» AND « outcome and process assessment, health care». First, the titles were reviewed, and then the abstracts and full texts of the selected articles were reviewed independently by two reviewers with the Abstrackr tool [17]. Any disagreements were resolved by consensus; MJ, MA, and JFH resolved any remaining disagreements.

After reading the full texts, we included the following studies in this analysis:

- studies reporting on IPC
- studies conducted in the primary care setting, involving primary care providers exclusively
- studies involving at least 2 different primary care providers, regardless of the type and level of collaboration (from a simple phone call to a multidisciplinary medical appointment).

The exclusion criteria were as follows:

- interventions involving multidisciplinary teams working between primary and secondary care
- the absence of a primary endpoint centred on patient health (studies focusing on economic outcomes, manuscripts reporting practices for declarative data only)
- the absence of a comparative design with a control group and statistical analysis (studies based on a before-after design involving the follow-up of only one cohort of patients were excluded)
- abstracts not respecting the IMRAD structure
- manuscripts not accessible in English.

One reviewer (MA) independently extracted data using a prepiloted form and was supervised by a second reviewer (CB), and the following data were collected: study country, pathology(ies) studied, intervention, the number of patients included, design, study duration, main outcome measures, and patient outcomes (selected on the basis of frequency of reporting and clinical relevance). For consistency and clarify of presentation, the results centred on patient outcomes are grouped within 3 categories in the remainder of the manuscript: 1- patients at cardiovascular risk, 2-polypathological and elderly patients, and 3- patients with mental health problems, chronic pain and unexplained complaints.

The quality of the studies was then assessed using the revised Downs and Black Checklist [18]. The checklist includes 27 items on reporting (10 items), external validity (3 items), internal validity (13 items), and power (1 item). Similar to others studies, the power item was modified regarding whether a power analysis was described (0 = not reported, 1 = reported). The maximum possible score is 28 for randomized studies and 25 for nonrandomized studies. Quality was categorized by using the following Downs and Black score ranges: strong (21-28), moderate (14-20), limited (7-13), and poor (≤ 7) [19].

## Results

### Selection and general description of the studies

**Fig. 1:**
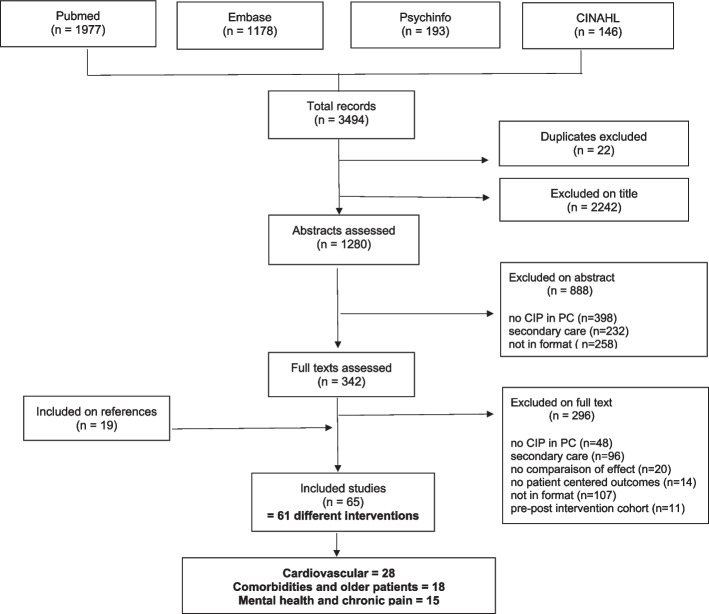
Flowchart

In total, 3494 titles, 1280 abstracts and 342 full-text papers were screened for eligibility using the inclusion and exclusion criteria (Fig. 1). Sixty-five papers were included in the review, comprising 61 interventions [20–84].

A large majority of the included studies were from North America (40) or Europe (13). The other studies were from Asia (5), Australia (2), and South America (1). Forty-three studies were prospective and randomized. Four studies were carried out over a period of more than 24 months [32, 34, 36, 64].

Depending on the studies, the number of patients included varied between 50 and 312,377: 5 studies included more than 5,000 patients, 37 studies included between 200 and 5,000 patients, and 19 studies included fewer than 200 patients.

### Constitution of the teams (Table 1)

**Table 1:** Number and type of health professionals involved in the intervention

**1GP + 1 other health professional**	**28**
*Pharmacist*	*14*
*Nurse*	*10*
*Psychologist*	*2*
*Health assistant*	*2*
**1GP + 2 or more different health professionals**	**33**
*Pharmacist*	*10*
*Nurse*	*27*
*Psychologist/psychiatrist*	*13*
*Health assistant*	*2*
*Dietician*	*10*
*Social worker*	*8*
*Medical specialist*	*7*
*Physiotherapist*	*5*
*Podiatrist*	*1*
*Occupational therapist*	*2*

### Pathologies

All the studies evaluating the effect of IPC on patients with chronic diseases: patients at cardiovascular risk (28 studies), elderly and/or polypathological patients (18 studies), or patients with mental health problems (12 studies). One study addressed chronic pain related to musculoskeletal disorders [78], and 2 other studies included patients with medically unexplained complaints [77, 82]. We chose to include these studies in the same paragraph and in the same table as patients with mental health difficulties. One study included in the polypathology group evaluated criteria for monitoring comorbidities (warfarin testing compliance, eye care compliance for diabetes, etc.) and cancer screening in the general population (mammograms and occult blood screenings) [67].

### Effect on patient variables

#### Patients at cardiovascular risk (Tables 2 and 3)

**Table 2:** Studies on the effect of interprofessional collaborations in patients at vascular risk

Author year	Region	Design	Population / Pathology	Intervention	Number	Number of type of professionals	Duration
Agarwal 2019 [20]	North America	P / Rd	Diabetes	Diabetes education and self-management by a multi-modal training program	50	> 2	< 12 months
Barceló 2010 [21]	South America	P / Rd	Diabetes	Multidisciplinary care: professional training and diabetes education for patients	307	> 2	12—24 months
Benedict 2018 [22]	North America	R / NRd	Diabetes	Adding clinical pharmacists to an integrated health care team	1960	> 2	12—24 months
Carter 2009 [23]/ Chen 2013 [24]	North America	P / Rd C	Hypertension	Physician-pharmacist co-management	402	2	< 12 months
Carter 2015 [25]	North America	P / Rd C	Hypertension	Physician/pharmacist collaborative model	625	2	12—24 months
Carter 2018 [26]	North America	P / Rd C	Cardiovascular diseases	Physician/pharmacist centralized collaborative care	302	2	12—24 months
Chen 2010 [27]	North America	P / NRd	Diabetes / hypertension	Health coaches visits and calls / coordination with resident primary care physicians	146	2	12—24 months
Choi 2015 [28]	Asia	P / NRd	Brain stroke	Secondary stroke prevention programme	577	2	12—24 months
Chwastiak 2017 [29]	North America	R / NRd	Diabetes	Multi-condition collaborative care: assessment, education self-management, behavioural interventions, care coordination	634	> 2	12—24 months
Edwards 2012 [30]	North America	R/ NRd	Diabetes	Diabetes Assessment Service (DAS)/ collaboration with pharmacist	304	2	12—24 months
ElGerges 2020 [31]	Asia	P / Rd	Diabetes	Therapeutic patient education	100	> 2	< 12 months
Fokkens 2011 [32]	Europa	P / NRd	Diabetes	Structured care: multidisciplinary cooperation and patients + professionals education	1001	> 2	> 24 months
Furler 2017 [33]	Australia	P / Rd	Diabetes	Reconfigured role for primary care: practice nurses / interaction GPs	266	2	12—24 months
Heisler 2012 [34]	North America	P / Rd	Diabetes	Adherence and intensification of medications intervention by pharmacists	4100	2	> 24 months
Jameson 2010 [35]	North America	P / Rd	Diabetes	Pharmacist management in a community-based primary care group	103	> 2	12—24 months
Jiao 2014 [36] / Jiao 2015 [37]	Asia	P / NRd	Diabetes	Multidisciplinary risk assessment and management program	2496 / 18188	> 2	> 24 months
Manns 2012 [38]	North America	R / NRd	Diabetes	Care managed in a primary care network	154928	> 2	12—24 months
McAdam- Marx 2015 [39]	North America	R/ NRd	Diabetes	Clinical pharmacy services in community-based primary	697	> 2	12—24 months
Mousquès 2010 [40]	Europa	P / NRd	Diabetes	Collaboration nurses/ physicians / patient education	1684	2	< 12 months
Mundt 2015 [41]	North America	R/ NRd	Cardiovascular disease	Different primary care social networks, with different level of interaction	7457	> 2	12—24 months
Pape 2011 [42]	North America	P / Rd	Diabetes	Team-based care approach with electronic medical record	6963	2	12—24 months
Simpson 2011 [43] /Omran 2015 [44]	North America	P / Rd	Diabetes	Adding pharmacists to primary care team	260	> 2	12—24 months
Smith 2004 [45]	Europa	P / Rd	Diabetes	Education of participants practitioners, introduction of a community-based diabetes nurse specialist, protocols, and communication	183	> 2	12—24 months
Smith 2016 [46]	North America	P / Rd	Hypertension	Physician-pharmacist collaborative mode	169	2	< 12 months
Tahaineh 2011 [47]	Asia	P / Rd	Dyslipidaemia	Physician–pharmacist collaboration and patient education	152	2	< 12 months
Tobari 2010 [48]	Asia	P / Rd	Hypertension	Physician–pharmacist program of cooperation	132	2	< 12 months
Vitale 2020 [49]	North America	P / Rd	Diabetes	Teams of nurse and dietitian: self-management education	771	> 2	12—24 months
Weber 2010 [50]	North America	P / Rd C	Hypertension	Pharmacist-physician comanagement	175	2	< 12 months

*P* Prospective, *R* Retrospective, *Rd* randomised, *Rd C* Cluster randomised, *NRd* nonrandomised

**Table 3:** Effects of interprofessional collaborations in patients at vascular risk

Author year	Main Outcomes	Results		*Statistics*	
Agarwal 2019 [20]	Self-Efficacy for Diabetes scale at 4 months	I = 7,93 C = 7,06 Difference at 4 months = 0.65 95%CI [- 0.11—1.40]		NS	
Barceló 2010 [21]	Percent of patients at the target			Group comparison at the end	
	**HbA1c < 7%**	I = 27.6% to 39.3% C = 20.7% to 27.9%		*p* = 0.03	
	Chol-total < 200	I = 65.1% to 76.5% C = 54.1% to 58.6%		NS	
	BP ≤ 140/90	I = 73.4% to 75.1% C = 72.5% to 69.3%		NS	
	**Foot examination done**	I = 49.0% to 95.4% C = 46.8% to 21.6%		*p* < 0.01	
	**Eye examination done**	I = 10.2% to 73.0% C = 3.6% to 4.5%		*p* < 0.01	
	**Three or more treatment goals**	I = 16.6% to 69.7% C = 12.4% to 5.9%		*p* < 0.01	
Benedict 2018 [22]	HbA1c < 8% at 12 months	Adj. OR = 0.88 95%CI [0.72–1.07]		NS	
Carter 2009 [23] Chen 2013 [24]	Improvement in guideline adherence at 6 months	I = 22.4 C = 4.0 Adj. OR = 9.6 95% CI [-2.3—21.5]		NS	
	**Nb of patient with controlled blood pressure**	I = 63.9% C = 29.9% Ad. OR = 3.2 95% CI [2.0—5.1]		*p* < 0.001	
	**SBP variation at 6 months**	I = -20.7 C = -6.8 Ad.. effect = -12.0 95% CI [-24.0—0.0]		*p* < 0.05	
	DBP variation at 6 months	I = -9.7 C = -4.5 Ad. effect = -1.8 95% CI [-11.9—8.3]		NS	
	**Mean 24-h SBP**	I = 135.6 to 120.4 C = 137.0 to 131.8		Baseline NS / 6 months *p* < 0.001	
	**Patients with controlled SBP(%)**	I = 39.8 to 75.6 C = 35.4 to 50.0		Baseline NS / 6 months *p* < 0.001	
Carter 2015 [25]	**SBP at 9 months**	I = 131.6 C = 138.2		*p* = 0.002	
	**DBP at 9 months**	I = 76.3 C = 78.0		*p* = 0.005	
Carter 2018 [26]	**Evolution of Guideline Advantage Score**	I = 63.3% to 67.8% (p = 0.02) C = 64.7% to 63.1% (NS)		*p* = 0.07	
Chen 2010 [27]				Adj. *p*-value	
	**LDL-C measured**	I = 74.0% to 84.9% C = 56.2% to 72.9% Difference in change = -5.8%		*p* = 0.001	
	HbA1c measured	I = 86.9% to 88.9% C = 93.7% to 90.1% Difference in change = + 5.6%		NS	
	BP (proportion of patients at goal)	I = 48.7% to 56.5% C = 41.4% to 45.4% Difference in change = + 3.8%		NS	
	LDL-C (proportion of patients at goal)	I = 49.1% to 58.6% C = 52.5% to 58.8% Difference in change = + 3.2%		NS	
	HbA1C (proportion of patients at goal)	I = 26.7% to 36.7% C = 25.9% to 34.8% Difference in change = + 1.8%		NS	
Choi 2015 [28]	New stroke	I = 2.7% C = 0.8%		NS	
	Pré to post intervention:				
	**SBP**	I = 135.2 to 127.7 C = 135.7 to 134.5 (no difference at baseline)		I: *p* < 0.01 C: NS	
	DBP	I = 70.4 to 68.1 C = 73.5 to 72.1 (difference at baseline *p* < 0.01)		I: *p* < 0.01 C: *p* = 0.04	
	**HbA1c**	I = 7.2 to 6.5 C = 7.2 to 6.9 (no difference at baseline)		I: p < 0.01 C: *p* = NS	
	LDL-C	I = 3.4 to 2.8 C = 3.0 to 3.0 (difference at baseline *p* < 0.01)		I: *p* < 0.01 C: p = NS	
	Ex-smoker	I = 14. % To 18.8% C = 23.3% to 27.1% (no difference at baseline)		I: *p* < 0.01 C: *p* < 0 .01	
Chwastiak 2017 [29]	**Change in HbA1c**				
	**Change in SBP**	I = -0.9% C = -0,2%		*p* = 0.008	
	**Outpatient clinic visits**	I = -3 mmHg C = + 1,4 mmHG		*p* = 0.014	
	Emergency department visits	I = 14.5 C = 8.3		*p* < 0.001	
		I = 1.8 C = 1.5		NS	
Edwards 2012 [30]	Proportion of patients with:				
	**HbA1c measured**	I = 91,2% C = 76,7% OR 3,13 95%CI [1,52–6,46]		*p* = 0.0013	
	**LDL-C measured**	I = 95,6% C = 70,0% OR 9,26 95%CI [3,60–23,79]		*p* < 0.0001	
	**Foot exam**	I = 87,6% C = 47,6% OR 7,78 95%CI [4,18–14,48]		*p* < 0.0001	
	**Eye referral**	I = 85,8% C = 55,2% OR 5,29 95%CI [2,88–9,72]		*p* < 0.0001	
	**Pneumococcal vaccine**	I = 80,5% C = 37,6% OR 7,26 95%CI [4,19–12,59]		*p* < 0.0001	
	**Influenza vaccine**	I = 74,3% C = 50,0% OR 2,90 95%CI [1,75–4,78]		*p* < 0.0001	
	**Urine sample**	I = 75,2% C = 15,7% OR 17,08 95%CI [9,66–30,21]		*p* < 0.0001	
ElGerges 2020 [31]	HbA1c	Before: I = 8.40 C = 7.7 After: I = 6.8 C = 7.5		Comparison I-C: before *p* < 0.05 after *p* < 0.05	
	SBP	Before: I = 132.0 C = 129 After: I = 125.6 C = 129.0		Comparison I-C: before NS after NS	
	**DBP**	Before: I = 81.0 C = 82.2 After: I = 76.2 C = 81.2		Comparison I-C: before NS after *p* < 0.01	
	**DMSES** (Diabetes Management Self-Efficacy Scale)	Before: I = 5.02 C = 4.91 After: I = 8.28 C = 4.85		Comparison I-C: before NS after *p* < 0.01	
	**SDSCA** (Summary of Diabetes Self-Care Activities)	Before: I = 2.89 C = 2.67 After: I = 4.56 C = 2.48		Comparison I-C: before NS after *p* < 0.01	
Fokkens 2011 [32]	Difference after 1 year				
	**HbA1c**	I = 0,2 C = 0.2 Adj. OR = 1.8 95%CI[1.03–3.14]		*p* < 0.05	
	SBP	I = -2,7 C = 1.0 Adj. OR = 1.54 95%CI [0.99–2.38]		NS	
	**DBP**	I = -1,8 C = -0.4 Adj. OR = 2.13 95%CI [1.37–3.32]		*p* < 0.05	
	**LDL-C**	I = -0,2 C = -0.4 Adj. OR = 2.89 95%CI [1.47–5.69]		*p* < 0.05	
Furler 2017 [33]	**Change (from baseline to 12 months) in HbA1c**	I = -1,3% C = -0,6%		*p* < 0.001	
Heisler 2012 [34]	Relative change in SBP at 6 months	I =—8.9 C = -9.0 -0.18 [-0.77- 1.13]		NS	
Jameson 2010 [35]	HbA1C evolution	I = -1.50 C = -0.40		NS	
Jiao 2014 [36] / Jiao 2015 [37]	**Changes in HbA1c**	I = -0,11 C = 0.10		*p* < 0.01	
	**Observed cardiovascular events**	I = 1.21% C = 2.89%		*p* = 0.003	
	**Predicted 10-year cardiovascular risks Framingham**	I = -3.93 C = -1.87		*p* < 0.01	
	Time to first occurrence of a major diabetes-related complication:	Hazad ratio I versus C			
	**composite of 3 cardio-vascular diseases**	0.629 95%CI [0.554, 0.715]		< 0.001	
	**coronary heart disease**	0.570 95%CI [0.470—0.691]		< 0.001	
	**stroke**	0.652 95%CI [0.546—0.780]		< 0.001	
	**heart failure**	0.598 95%CI [0.446—0.802]		0.001	
	**all-cause mortality**	0.363 95%CI [0.308—0.428]		< 0.001	
Manns 2012 [38]	Rate of **admissions to hospital** or visits to **emergency** departments per 1000 patients/month	I = 1.58 C = 1.96 OR = 0.83 95%CI [0.64, 1.08]		*p* < 0.001	
McAdam-Marx 2015 [39]	**HbA1c at 18 months**				
	**Baseline HbA1c ≥ 7.0%:**	OR ad. -0.212 CI 95% [-0.401—-0.023]		*p* = 0.028	
	**Baseline HbA1c ≥ 8.0%:**	OR ad. -0.381 CI 95% [-0.616 à -0.146]		*p* = 0.002	
Mosques 2010 [40]	Realization rates for:				
	**HbA1c**	OR (Ref = control group)		*p* = < 0.0001	
	**Microalbuminuria**	I = 1.868		*p* = < 0.0001	
	Funduscopy	I = 6.716		NS	
	**Creatinemia**	I = 1.207		*p* = < 0.0001	
	**ECG**	I = 2.761		*p* = < 0.0001	
	**Lipid check-up**	I = 2.547		*p* = < 0.0001	
		I = 2.154			
Mundt 2015 [41]		Full Model for Team social network			
		Density:	Centralization	Density	Centralization
	BP < 130/80 mmHg	OR = 1.15 [0.99–1.34]	OR = 1.03 [0.85–1.25]	NS	NS
	LDL-c < 100 mg/Dl	OR = 1.14 [1.00–1.31]	OR = 0.93 [0.79–1.08]	NS	NS
	Nb of urgent care visits	OR = 0.95 [0.55- 1.66]	OR = 1.20 [0.79–1.81]	NS	NS
	Nb of emergency department visits	OR = 0.98[0.50–1.89]	OR = 1.33 [0.83–2.13]	NS	NS
	**Nb hospital visit days**	OR = 0.62 [0.50–0.77]	OR = 1.45 [1.09–1.94]	*p* < 0.001	*p* < 0.001
Pape 2011 [42]	**Patient target: LDL-C < = 100 mg/dL (%)**	I = 78% C = 50%		*p* = 0.003	
	**LDL-c**	I = 83 vs 95		< 0.001	
Simpson 2011 [43] Omran 2015 [44]	** ≥ 10% decrease in SBP at 1 year**	I = 37% C = 23% OR 1.91 95% CI [1.11–3.28]		*p* = 0.02	
	** > = 1 hypertensive treatment optimization**	I = 42% C = 26% OR = 1.63 95% CI [1.08–2.46]		*p* = 0.016	
Smith 2004 [45]	HbA1c	I = 7.0% C = 6.7%		NS	
Smith 2016 [46]	**SBP 0 to 9 months**	I = 149 to 132 C = 150 to 141		Baseline NS / 9 months *p* = 0.036	
	SBP 0 to 9 months	I = 84 to 75 C = 79 to 73		Baseline NS / 9 months NS	
	BP control at 9 months	I = 34,2% C = 25,9% Ad. OR = 1.92 95% CI [0.33–11.2]		NS	
Tahaineh 2011 [47]	**Percent of patients at their LDL-c target**	I = 94.5% C = 71.2%		*p* < 0.001	
Tobari 2010 [48]	variation at 6 months:				
	At office:				
	SBP	I = -2.4 C = -0.9		NS	
	DBP	I =—2.3 C = -3.1		NS	
	At home morning				
	SBP	I = -2.9 C = -1.2		NS	
	**DBP**	I = -3.3 C = -1.4		*p* = 0.04 CI [-5.5; -0.1]	
	**BMI**	I = -0.4 C = -0.0		*p* = 0.008 CI [− 0.7; − 0.1]	
Vitale 2020 [49]	Realization of	Overall effect size in OR			
	HbA1c	= 1.15		NS	
	BP	= 1.06		NS	
	**Diabetes management visit**	= 1.22		*p* = 0.02	
	LDL-C	= 0.87		NS	
	**Foot exam**	= 1.19		*p* = 0.05	
Weber 2010 [50]	**Change in 24-h mean ambulatory SBP and DBP (mmHg)**	Reduction SBP I = -14.1 C = -5.5		*p* < 0.001	
		Reduction DBP I = -6.8 C = -2.8		*p* < 0.001	

*I* group intervention (= IPC), *C* Control group, *OR* odds-ratio, *CI* Confidence interval, *Adj.* Adjusted, *NS* non-significant, *DBP* Diastolic Blood Pressure, *SBP* Systolic Blood Pressure

The 28 studies addressing cardiovascular risk focused particularly on diabetes (18 studies), hypertension (5 studies), overall cardiovascular risk (4 studies), or dyslipidaemia (1 study). The most common primary endpoints were glycated haemoglobin levels (14 studies), blood pressure (14 studies) and LDL-c or total cholesterol levels (9 studies). Three studies had a real morbidity criterion (cardiovascular events) as the primary endpoint, and 3 other studies assessed the number of visits to the emergency department. Fifteen studies described IPC with pharmacists, and 15 described IPC with nurses.

Interventions around cardiovascular pathologies were mainly based on team-based patient education or doctor/pharmacist collaboration (medication review, blood pressure monitoring, frequent contact about treatment by phone, through the patient's file or concertation meetings).

Of the 28 studies focusing on cardiovascular risk, five reported no significant results for their main endpoints. Benedict's study [22] which included 1960 patients, showed effects on the secondary endpoints, particularly in the short term.

The Heisler [34] cluster randomized trial focused on physician/pharmacist collaboration. It included 4100 patients but failed to show positive effects on blood pressure at 6 months, and only short-term secondary results showed a 2.4 mmHg improvement in blood pressure related to the intervention. Both groups (control and intervention) showed improvement during the study. The nonrandomized study by Manns [38] including 150,000 diabetic patients was able to show the effectiveness of the management of diabetic patients in the primary care network, with a reduction in the number of hospital and emergency department visits. Secondary analyses also showed an improvement in ophthalmological follow-up and glycaemic control. Jiao [36, 37] also showed an improvement in HbA1c levels and in the occurrence of cardiovascular events (from 2.89% to 1.21%) for the group participating in a diabetes monitoring program. These 2 studies offered network support that included many diabetes professionals: podiatrists, nurses, and dietitians.

Of the 15 studies analysing effects on glycated haemoglobin levels as the primary outcome, 10 reported positive results [21, 28–33, 36, 39, 40]. Conversely, 5 studies reported no significant effect on this variable as the primary outcome measure [22, 27, 35, 45, 49].

Among the 15 studies analysing effects on blood pressure, 10 reported positive results [23, 26, 28, 29, 31, 32, 43, 46, 48, 50]. In 3 studies the positive results were only for diastolic blood pressure, with no effect on systolic blood pressure, and one article found an improvement only in systolic pressure without improvement in diastolic blood pressure. Five studies concluded that there was no effect on blood pressure [21, 27, 34, 41, 49].

Among the 9 studies analysing an effect on cholesterol levels, 6 reported positive results while 3 concluded that there was no effect [21, 41, 49].

#### Elderly and/or polypathological patients (Tables 4 and 5)

**Table 4:** Studies on the effect of interprofessional collaborations in elderly and/or polypathological patients

Author year	Region	Design	Population / Pathology cible	Intervention	Number	Number of type of professionals	Duration
Aigner 2004 [51]	North America	R / Rd	Elderly people	Collaboration nurse practitioners / physicians	203	2	12—24 months
Boult 2008 [52]/Leff 2009 [53]	North America	P / Rd	Elderly people and comorbidities	Guided Care: assessment, care guide, action plan, monthly monitoring, patient and family caregivers’ education, coordination of cares	904	2	12–24 months
Boult 2011 [54]	North America	P / Rd	General population	Guided Care: assessment, care guide, action plan, monthly monitoring, patient and family caregivers’ education, coordination of cares	850	2	12—24 months
Boyd 2010 [55]	North America	P / Rd	Elderly people and comorbidities	Guided Care: assessment, care guide, action plan, monthly monitoring, patient and family caregivers’ education, coordination of cares	2391	2	12—24 months
Brown 2003 [56]	Europa	P / NRd	Elderly people	Integrated health and social care	393	> 2	12—24 months
Burns 2000 [57]	North America	P / Rd	Elderly people (veterans)	Interdisciplinary primary care team: assessment and management	128	> 2	12—24 months
Dolovich 2019 [58]	North America	P / Rd	Elderly people	Person-centred and team-based primary care intervention, including new health care elements	312	> 2	< 12 months
Hogg 2009 [59]	North America	P / Rd	Comorbidities	Multidisciplinary team: Physicians, nurse practitioners, pharmacists	241	> 2	12—24 months
Lenaghan 2007 [60]	Europa	P / Rd	Elderly people	Physician–pharmacist collaboration and patient education	136	2	< 12 months
Lin, 2014 [61]	North America	P / Rd	Depression / diabetes/ coronaropathy	Patient-centred collaborative care program	214	> 2	12—24 months
Matzke 2018 [62]	North America	P / NRd	Comorbidities	Inclusion of clinical pharmacists in this physician– pharmacist collaborative care–based patient-centred medical home model	4960	> 2	12—24 months
Melis 2008 [63]	Europa	P / Rd PC	Elderly people	Multidisciplinary community intervention model: nurse home visits, GP inclusion	151	> 2	< 12 months
Riverin 2017 [64]	North America	R/ NRd	Elderly people and comorbidities	Multidisciplinaryy team-based primary care practice	312377	2	> 24 months
Sellors 2003 [65]	North America	P / Rd	Elderly people	Collaboration pharmacist/physician	889	2	< 12 months
Sommers 2000 [66]	North America	P / Rd	Elderly people	Interdisciplinary collaborative practice intervention	543	> 2	12—24 months
Taplin 1998 [67]	North America	P / NRd	General population	Reorganization of a care team around population-based care	1460	> 2	12—24 months
Van Lieshout 2018 [68]	Europa	P / Rd	Elderly people	Interdisciplinary multicomponent intervention program: a medication review, physical fitness, social skills, and nutrition	290	> 2	12—24 months
Wolff 2010 [69]	North America	P / Rd	Comorbidities	Guided Care on Family caregivers: assessment, care guide, action plan, monthly monitoring, patient and family caregivers’ education, coordination of cares	196	2	12—24 months

*P* Prospective, *R* Retrospective, *Rd* randomised, *Rd PC* Pseudo cluster randomised, *NRd* nonrandomised

**Table 5:** Effects of interprofessional collaborations in elderly and/or polypathological patients

Author year	Main Outcomes	Results	Statistics
Aigner 2004 [51]	Number of visits to the emergency department per year	I = 1.3 C = 1.1	NS
	Number of hospital admissions per year	I = 0.6 C = 0.5	NS
	Completion of mandated progress visits and histories	I = 4.6 C = 4.5	NS
	**Number of acute visits per year**	I = 3.0 C = 1.2	*p* < 0.0001
	Average number of medications	I = 6.4 C = 6.2	NS
Boult 2008 [52] Leff 2009 [53]	**PACIC (Patient Assessment of Chronic Illness Care)**	I = 17.4 C = 8.5; Adj. OR = 2.03 [1.22; 3.39]	*p* = 0.006
	Hospital admissions	I = 0.75 C = 0.96 Adj.OR = 0.83 95%CI [0.64, 1.08]	NS
	Emergency department visits	I = 0.36 C = 0.43 Adj. OR = 0.85 95%CI [0.62, 1.18]	NS
	Primary care physician visits	I = 9.85 C = 10.13 Adj. OR = 1.00 95%CI [0.88, 1.14]	NS
Boult 2011 [54]	Hospital admissions	I = 0.57 C = 0.61 Adj.effect 0.85 [0.61–1.19]	NS
	30-Day readmissions	I = 0.09 C = 0.16 Adj.effect 0.51 [0.23–1.15]	NS
	Hospital days	I = 3.36 C = 3.90 Adj.effect 0.79 [0.53- 1.19]	NS
	**Skilled nursing facility admissions**	I = 0.13 C = 0.23 Adj.effect 0.53 [0.31–0.89]	S
	**Skilled nursing facility days**	I = 2.09 C = 4.09 Adj.effect 0.48 [0.28–0.84]	S
	Emergency department visits	I = 0.37 C = 0.44 Adj.effect 0.83 [0.56–1.21]	NS
	Primary care visits	I = 9.35 C = 8.59 Adj.effect 1.08 [0.90–1.29]	NS
	Specialist visits	I = 0.63 C = 0.32 Adj.effect 0.93 [0.75–1.15]	NS
Boyd 2010 [55]	**PACIC (Patient Assessment of Chronic Illness Care)**	I = 3.14 C = 2.85 Adj. Effect = 0.20 [0.07, 0.33]	*p* = 0.002
Brown 2003 [56]	% People living independently at 18 months	I = 66% C = 62%	NS
Burns 2000 [57]	Death	I = 16.7% C = 27.9%	NS
	Group difference		
	**GHP (Health perception)**	0.011	0.001
	**Clinic visits**	0.877	0.019
	Hospitalizations	0.177	NS
	Katz ADL (functional status)	0.078	NS
	**IADL (functional status)**	0.701	0.006
	**CES-D (quality of life)**	0.010	0.003
	**MMS**	0.212	0.025
Dolovich 2019 [58]	Goal attainment scaling	I = 57.79 C = 58.94 Adj. Effect -1.50 95% CI [− 6.51 to 3.50]	NS
Hogg 2009 [59]	**Variation quality of care-chronic disease**	I = 0.098 C = 0.008 Difference = 0.091 95%CI[0.037—0.144]	*p* = 0.0013
	**Variation quality of care-prevention**	I = 0.126 C = -0.056 Difference = 0.181 95%CI[0.108—0.255]	*p* < 0.001
Lenaghan 2007 [60]	Total non-elective hospital admissions within 6 months	I = 21 C = 20	NS
Lin, 2014 [61]	Unfavourable control at baseline / C-I at 2 years		
	HbA1c	C-I = -0.3 Ad. Effect size = − 0.88 95%CI [− 0.99—0.38]	NS
	LDL-C	C-I = -9.1 Ad. Effect size = − 0.93 95%CI [− 28.7- 10.5]	NS
	SBP	C-I = -3.1 Ad. Effect size = − 0.70 95%CI [− 11.9—5.7]	NS
	Favourable control at baseline / C-I at 2 years		
	HbA1c	C-I = 0.27 Ad. Effect size = − 1.26 95%CI [− 0.16—0.70]	NS
	LDL-C	C-I = 3.7 Ad. Effect size = 0.73 95%CI [− 6.2 -13.6]	NS
	SBP	C-I = 2.1 Ad. Effect size = 0.76 95%CI [− 3.4—7.7]	NS
Matzke 2018 [62]	Mean reduction:		
	**HbA1c**	I = 0.46 95%CI [0.33—0.58] C = 0.08 95%CI [–0.02—0.18]	*p* < 0.0001
	**SBP**	I = 6,28 95%CI [4.88—7.68] C = 1,05 95%CI [–0.20—2.30]	*p* < 0.0001
	**DBP**	I = 2,69 95%CI [1.99—3.39] C = 1,23 95%CI [0.51—1.94]	*p* = 0.0071
	LDL-C	I = 3,72 95%CI [0.88—6.57] C = 4,15 95%CI [1.66—6.64]	NS
	Chol-total	I = 5,08 95%CI [1.67—8.49] C = 5,34 /95%CI [2.43—8.25]	NS
Melis 2008 [63]	**GARS-3** (Groningen Activity Restriction Scale-3)	OR = -2.2 95% CI [-4.2 to 0.3]	*p* < 0.05
	**MOS-20 MH** (Mental Health of the Medical Outcome Study)	OR = 5.8 95% CI [0.1 to 11.4]	*p* < 0.05
Riverin 2017 [64]	Hospital readmission within 90 days after discharge	I = 136.3 C = 140.6 Ad. OR = 1.2 CI 95% [-2.1—4.5]	NS
Sellors 2003 [65]	Daily units of medication taken	I = 8.0 C = 7.9	NS
Sommers 2000 [66]	*Medical service utilization*:		
	**Hospital admissions per patient**	I = 0.18 C = -0.02 OR = 0.63 95% CI [0.41–0.96]	*p* = 0.03
	**Within 60 days readmissions**	I = 5.4 C = -2.0 OR = 0.26 95% CI [0.08—0.84]	*p* = 0.03
	**Office visits**	I = 0.5 C = -1.5 OR = 0.85	*p* = 0.003
	Emergency department visit	I = -0.56 C = 1.2	NS
	Home care visits	I = 2.6 C = 1.8	NS
	*Health status measures:*		
	**Social activities count**	I = -0.3 C = 0.2 95% CI [0.02–1.0]	*p* = 0.04
	**Symptom scale**	I = 1.0 C = -0.5 95% CI [-3.2—0.16]	*p* = 0.08
	**SF-36**	I = 0.1 C = 0.0 95% CI [-0.27—0.02]	*p* = 0.08
	HAQ /GDS/ Medication count / Nutrition checklist	NS	NS
Taplin 1998 [67]		Differences Study/ Surrounding / GHC population	
	**Mammogram**	Study group rate improved and faster	< 0.01
	**Occult blood screening**	Study group rate improved and faster	*p* < 0.017
	Warfarin testing compliance	No improvement for study group	NS
	Eye care compliance diabetes	No improvement for study group	NS
Van Lieshout 2018 [68]	Activity of daily living (ADL) measured with the Katz-6	Adj. Katz-6 score OR = 0.96 95%CI: [0.39–2.35]	NS
Wolff 2010 [69]	At 18 months:		
	Caregiver strain CSI score	I-C = -0.38 Adj.effect size = -0.08 95% CI [− 0.37—0.20]	NS
	Depression CES-D score	I-C = 1.42 Adj. effect size = 0.23 95% CI [− 0.06—0.51]	NS
	**Quality of chronic care (PACIC- Aggregate quality)**	I-C = 0.40 Adj. effect size = 0.47 95% CI [0.15—0.78]	*p* < 0.001
	Productivity loss (WPAI:CG)		
	Regular activity	I-C = − 0.05 Adj. effect size = − 0.26 95% CI [− 0.74—0.22]	NS
	Work productivity	I-C = 0.00 Adj. effect size = 0.01 95% CI [− 0.28—0.30]	NS

*I* group intervention (= IPC), *C* Control group, *OR* odds-ratio, *CI* Confidence interval, *Adj.* Adjusted, *NS* non-significant, *DBP* Diastolic Blood Pressure, *SBP* Systolic Blood Pressure

The results of studies on the effect of IPC on the care of elderly or polypathological patients are inconsistent. Of the 18 studies included, 10 reported significant positive results, of which 8 were randomized controlled trials. Fifteen of these studies included doctors and nurses, after which pharmacists were the professionals most involved in care. The retrospective study by Riverin [64] associated nurses with doctors and was based on a population of 312,377 patients. The study did not demonstrate any improvement in the primary outcome measure: hospitalization 3 months after hospital discharge. It showed only a short-term decrease in the number of emergency room visits and deaths (fewer than 4 deaths per 1000 treated) in the group receiving the IPC intervention.

Eight studies did not show the effectiveness of their intervention on their primary outcome measure or variable. Eight randomized trials had documented effects. Three randomized trials [52, 55, 69] showed that the quality of care received by elderly patients was perceived as better when care was provided within the framework of a formalized collaboration among health professionals. The measurement tool in these 3 trials was the Patient Assessment of Chronic Illness Care (PACIC). The same type of result was observed in a study using the Quality of Care for Chronic Disease Management score [59].

With regard to functional abilities and patient symptoms, the randomized trial by Burns [57] showed beneficial effects of collaborative outpatient practices on patient variables relating to addiction (IADL, MMSE, maintenance of social activity) or mental health (CES-depression, general well-being, life satisfaction). The effect of IPC on hospital readmissions varied greatly among studies. Sommers et al. [66] reported a significant decrease in the number of admissions to the hospital or intensive care unit. Riverin et al. reported a decrease in the use of emergency rooms and a decrease in mortality (secondary outcome measure of the study) [64]. Conversely, some randomized trials concluded that there was no impact on their primary outcome measure [51, 53, 57, 60].

The only study focusing on cancer screening by mammograms and occult blood screenings in the general population showed better follow-up for patients followed by a health care team [67].

#### Patients with symptoms of mental or physical distress (Tables 6 and 7)

**Table 6:** Studies on the effect of interprofessional collaborations in patients with symptoms of mental or physical suffering

Author year	Region	Design	Population / Pathology	Intervention	Number	Number of type of professionals	Duration
Adler 2004 [70]	North America	P / Rd	Depression	Pharmacist intervention: assessment, patient education, communication with professionals	533	> 2	< 12 months
Aragonès 2019 [71]	Europa	P / Rd	Depression and MSDs	Care managed in a primary care team and psychoeducational programme	328	2	12—24 months
Areán 2007 [72]	North America	P / Rd	Depression	Collaborative care: physicians / psychiatrist / specialized nurse / psychologist	1801	> 2	12—24 months
Chan 2011 [73]	Europa	P / Rd	Anxiety and depression	Multidisciplinary team consultation	94	> 2	12—24 months
Engel 2016 [74]	North America	P / Rd	PTSD and depression	Centrally Assisted Collaborative Telecare	666	> 2	12—24 months
Finley 2002 [75]	North America	P / NRd	Depression	Collaborative pharmacy practice model including pharmacy specialists	220	2	< 12 months
Finley 2003 [76]	North America	P / Rd	Depression	Collaborative care emphasizing the role of pharmacist / patient education	125	2	< 12 months
Kolk 2004 [77]	Europa	P / Rd	Medically unexplained symptoms	Psychological intervention by a qualified therapist + Physicians	98	2	12—24 months
Marklund 1999 [78]	Europa	P / NRd	MSDs	Assessment, and adapted interventions / meetings occupational therapist, GP, and physiotherapist	138	> 2	< 12 months
Morgan 2013 [79]	Australia	P / Rd	Depression by diabetes or cardiopathy patients	Practice nurse and GP every 3 months: evaluation and management	317	> 2	12—24 months
Petersen 2014 [80]	Europa	P / Rd	Depression	Collaborative care intervention with Chronic Care Model	626	2	12—24 months
Rollman 2005 [81]	North America	P / Rd	Anxiety	Telephone-based collaborative care	191	2	12—24 months
Schaefert 2013 [82]	Europa	P / Rd C	Medically unexplained symptoms	Collaborative group intervention: Professional’s training / interpersonal approach of psychodynamically based therapy	304	> 2	12—24 months
Sherbourne, 2001 [83]	North America	P / Rd	Depression	Quality improvement (QI) interventions for depression to primary care practices	1299	> 2	12—24 months
Simon 1998 [84]	North America	P / Rd	Depression	Patient education, on-site: mental health treatment, adjustment of antidepressant medication, behavioural activation, and monitoring of medication adherence	156	> 2	< 12 months

*P* Prospective, *R* Retrospective, *Rd* randomised, *Rd PC* Pseudo cluster randomised, *NRd* nonrandomised, *MSDs* Musculoskeletal disorders, *PTSD* Post traumatic stress disorder

**Table 7:** Effects of interprofessional collaborations in patients with symptoms of mental or physical distress

Author year	Main Outcomes	Results	Statistics
Adler 2004 [70]	**Antidepressant use rates at 6 months**	I = 57,5% C = 46,2%	*p* = 0,025
	Modification of the Beck Depression Inventory (BDI) at 6 months	I = 17.7 C = 19.4	NS
Aragonès 2019 [71]	Depression at 12 months		
	**Remission rate (HSCL-20 < 0.5)**	I = 20.1% C = 11.1% OR = 2.13 95% CI [0.94—4.85]	*p* = 0.070
	**Response to treatment (50% reduction HSCL-20)**	I = 39.6% C = 20.7% OR = 2.74 95% CI [1.12—6.67]	*p* = 0.027
	Pain at 12 months		
	Response to treatment (30% reduction BPI)	I = 18.7% C = 18.5% OR = 1.02 95% CI [0.46—2.26)	NS
Areán 2007 [72]	Patients at 12 months:	NOT POOR(NP) / POOR (P) patients	
	Use of antidepressant (%)	NP:I = 65 C = 49 Ad. OR = 2.17 95%CI [1.53—3.08]	*p* < 0.001
	Use of psychotherapy (%)	P:I = 68 C = 48 Ad. OR = 3.25 95%CI[2.14—4.96]	*p* < 0.001
	Depressive symptoms = SCL-20	NP:I = 44 C = 16 Ad. OR = 4.33 95%CI[3.14—5.97]	*p* < 0.001
	Health-related functional impairment = General health self-ratings	P:I = 40 C = 15 Ad. OR = 4.16 95%CI[2.52—6.85]	*p* < 0.001
		NP:I = 0.95 C = 1.36 Ad. OR = –0.41 95%CI[–0.49—–0.33]	*p* < 0.001
		P:I = 1.07 C = 1.45 Ad. OR = –0.39 95%CI[–0.50—–0.27]	*p* < 0.001
	PCS-12	NP:I = 3.06 C = 3.38 Ad. OR = -0.32 95%CI[–0.43—–0.21]	*p* < 0.001
		P:I = 3.40 C = 3.69 Ad. OR = -0.29 95%CI[–0.45—–0.12]	*p* < 0.001
		NP:I = 41.74 C = 39.88 Ad. OR = 1.67 95%CI[0.78—2.55]	*p* < 0.001
		P:I = 38.99 C = 37.76 Ad. OR = 1.46 95%CI[0.33—2.60]	*p* < 0.001
Chan 2011 [73]	**HADS (Hospital Anxiety and Depression Scale)**		
	**6 months**	I = 21.5 C = 17.5	0.061
	12 months	I = 19.5 C = 17.9	NS
Engel 2016 [74]	**Posttraumatic Diagnostic Scale (PDS) at 12 months**	I = -6.07 C = -3.54 OR 1.62 95%CI [1.08–2.43]	*p* = 0.02
	**Symptom Checklist Depression Scale (SCL-20) at 12 months**	I = -0.56 C = -0.31 OR 1.65 95%CI [1.13–2.42]	*p* = 0.01
Finley 2002 [75]	**Medication possession ratios**	I = 0.811 C = 0.659	*p* < 0.005
	**Variation of the nb of primary care visits**	I = -39.4% C = -12.2%	*p* < 0.007
Finley 2003 [76]	**Compliance early phase**	I = 76% C = 60% OR 2.11, 95%CI [ 0.97–4.58]	*p* = 0.057
	**Compliance continuation phase**	I = 67% C = 48% OR 2.17, 95%CI [1.04–4.51]	*p* = 0.038
	**MPR (medication possession ratio) at 3 months**	I = 0.92 C = 0.89	*p* = 0.48
	**MPR at 6 months**	I = 0.83 C = 0.77	*p* = 0.26
	**Change of antidepressants**	I = 19% C = 4%	*p* = 0.016
	**Resource utilization**	I = 5% C = 24%	*p* = 0.54
Kolk 2004 [77]	Pre-test to 12 months		
	Self-reported	I = 27,77 to 19.9 C = 25.19 to 21.00	NS
	unexplained symptoms	I = 22.55 to 15.39 C = 20.44 to 13.56	NS
	anxiety	I = 38.90 to 25.93 C = 34.56 to 23.12	NS
	depression		
	Registered	I = 4.39 to 1.95 C = 2.73 to 0.87	NS
	unexplained symptoms	I = 1.88 to 1.20 C = 1.47 to 1.53	NS
	explained symptoms	I = 4.95 to 3.39 C = 3.80 to 2.93	NS
	nb of consultations		
Marklund 1999 [78]	**Number of sick days**	Intervention group 63.8 / control group 92.8	0.006
Morgan 2013 [79]	**PHQ-9 (Ancova)**	I = 14,4 to 8,7 C = 15,1 to 10,8	*p* = 0,047
Petersen 2014 [80]	**PACIC = Patient Assessment of Chronic Illness Care**	I = 3.12 C = 2.86	*p* = 0.019
	Morisky patient self-report scale (Medication adherence)	I = 2.59 C = 2.65	NS
	Prescribed antidepressant medications	I = 60.2% C = 55.1%	NS
	Visits to the family physician	I = 15.96 C = 14.46	NS
	Visits to the mental health specialists	I = 3.01 C = 0.94	NS
Rollman 2005 [81]	**Hamilton Anxiety Rating Scale**	-3,6 [-6,4; -0,8] / effect size (95% CI): 0,38 [0,09; 0,67]	*p* = 0,01
Schaefert 2013 [82]	At 12 months quality of life:		
	Change in PCS (physical health part of SF-36)	I = 44.56 C = 44.14 Ad. OR = 0.32 95%CI[–1.20—1.84]	NS
	**Change in MCS (mental health part of SF-36)**	I = 46.59 C = 42.09 Ad. OR = 2.30 95%CI [0.34—4.26]	*p* = 0.0226
Sherbourne, 2001 [83]	Clinical depression at 2 years (CIDI)	I (meds) = 39%, I(therapy) = 31%, C = 34%	NS
Simon 1998 [84]	Unable to work due to illness	OR 0.60 [0.40, 0.91]	NS
	**Had to change work due to illness**	OR 0.80 [0.49, 1.33]	NS
	Cut down on activities due to illness	**OR 0.68 [0.46, 0.99]**	S
	**Rating health fair or poor**	OR 0.94 [0.68, 1.29]	NS
	Somatic symptoms	**OR 0.69 [0.48, 0.98]**	S
	Pain symptoms	OR 0.83 [0.63, 1.09]	NS
	Missing work/school	OR 1.25 [0.52, 3.09]	NS
	Restricting daily activities	OR 1.04 [0.20, 5.33]	NS
		OR 1.14 [0.38, 3.40]	NS

*I* group intervention (= IPC), *C* Control group, *OR* odds-ratio, *CI* Confidence interval, *Adj.* Adjusted, *NS* non-significant, *DBP* Diastolic Blood Pressure, *SBP* Systolic Blood Pressure

Among the 12 studies addressing mental health, the outcome measures sometimes included depression (10 studies), anxiety (1 study), or posttraumatic stress (1 study). Eleven studies were prospective and randomized, and 2 randomized trials included more than 1200 patients [72, 83]. Only one study did not show a significant result on the primary outcome measure [83], which was the clinical depression score at 2 years.

The evaluation of the effect of IPC treatment on psychological disorders involved various tools: the Hamilton Anxiety and Depression Scale [81], the Hospital Anxiety and Depression Scale [73], the Beck Depression Inventory (BDI) [70], the Symptom checklist-core depression (SCL) [71, 72, 74], the PACIC [80], the Composite International Diagnostic Validity [83], the HSCL [71], and the Patient Health Questionnaire [79]. Often, the studies did not use validated scores but simply used the rates of cure, recourse to care or therapy use [70–72, 75, 76, 84].

A positive effect of the IPC intervention on patients with psychological disorders was reported in 10 studies, at least in the short term (6 months). There were no significantly positive results for 2 studies [83, 84], including that of Sherbourne, which was the only study to assess depression at 2 years. Chan's study [73] showed an improvement in the HAD score at 6 months in the intervention group, but this effect was no longer statistically significant at 12 months.

Arean et al., Engel et al. and Aragonès et al. reported an improvement in the SCL-20 score [71, 72, 74]. Four articles reported the effect of collaboration on medication compliance in depressed patients [70, 72, 75, 76], while Petersen concluded that there was no effect on compliance with these treatments [80].

Two studies were only interested in medically unexplained symptoms: that of Kolk [77] did not report a positive effect, and that of Shaefert [82] only reported an improvement in the "mental health" component of the quality of life score (SF-36) but no improvement in physical symptoms or care utilization at 12 months.

A study on musculoskeletal disorders showed that multiprofessional management involving general practitioners, occupational therapists, physiotherapists and rheumatologists significantly reduced the number of days off work: 63.8 days in the intervention group versus 92.8 days in the control group [78].

### Assessment of study quality

The studies had an average quality score of 17 points (out of 28 points) using the revised Down’s and Black Checklist [min = 9, max = 21]. Eight studies had a high-quality score, 41 had a moderate-quality score, 12 had a limited-quality score (between 9 to 14 points), and no studies had a poor-quality score (< 9).

## Discussion

The studies that assessed the effect of IPC on patient outcomes could be grouped into 3 categories, depending on whether the patients 1) were at cardiovascular risk, 2) were elderly and/or had polypathology, or 3) had mental or physical disorders. One study also aimed to improve prevention care. Our review of the literature did not find any studies that evaluated the effect of IPC on patient outcomes in the following fields: orthopaedics and the musculoskeletal system, cancer care, paediatrics, current infectious diseases, and patient health monitoring. Only one study analysed cancer screening in addition to monitoring comorbidities. The positive effect of IPC on patient outcomes has been widely described in patients at vascular risk. Of 28 studies, only 5 reported no significant effect on their primary outcome measures. For the 2 other categories (elderly and/or polypathological patients, patients with physical or mental disorders), the reported effects varied from one study to another. Among 18 studies that assessed the effect of IPC on the outcomes of elderly or polypathological patients, only 10 studies reported positive effects on the primary outcome measure, and 11 out of 12 reported positive effects in the category of patients with mental disorders. In this last category, 10 studies focused on patients with depressive syndrome. The majority of studies reported clinical improvement in patients. The majority of the proposed interventions relied on 3 main health professionals: general practitioners, pharmacists and nurses. Other professions were less frequently included in the studies.

In general, studies evaluating the effect of IPC are difficult to compare since the interventions are often very different, and the designs and the evaluation criteria vary, making it impossible to conduct a meta-analysis of the data. The number of studies is often very limited: many fields of care have not been the subject of any study. Some pathologies are only approached in a very isolated way, e.g., chronic pain and anxiety disorders. Finally, for the two topics best covered (cardiovascular risk and depressive syndrome), the differences among the studies remain significant and limit the opportunity to aggregate the results. It is notable that the judgement outcomes are also very different. Compliance [44], patient satisfaction [42], or improvement in blood pressure or HbA1c levels [25, 29] cannot be compared. Finally, even when the studies analysed a bioclinical measure such as HbA1C levels, the authors chose different judgement criteria: the rate of prescription of an examination [51], the examination completion rate [30, 40, 49], the variation of the result over time or the rate of patients reaching their objective for this measure [22]. This complicates the comparison of studies. Smith did not show any effectiveness of his intervention on the HbA1c level in diabetic patients (the primary outcome measure) but showed that the proportion of patients who carried out the recommended monitoring examinations had increased [45]. For the 15 studies evaluating the effect of ICP on arterial hypertension, the diversity of judgement outcomes still remains significant, depending on whether the authors chose to analyse the mean systolic blood pressure value in the intervention arm [29], the change in systolic blood pressure over one year [34, 43], the change in diastolic blood pressure over one year [36], the proportion of patients with controlled systolic blood pressure (less than 140 mmHg) [43], the proportion of patients for whom the systolic blood pressure was measured (care procedure indicator) [26], the proportion of patients for whom an anti-hypertensive treatment dose adjustment was carried out [44], the evolution of the mean 24-h SBP [24], or the evolution of the SBP at home [48].

The clinical impact of the effects observed can also be questioned. For example, the study by Heisler [34], a large randomized study (4100 patients), showed a reduction of 2.4 mmHg in blood pressure thanks to the help of the pharmacist, which is clinically limited. With regard to depression, one can wonder about the benefit of a 3.6-point reduction in the Hamilton score for patients, even if the result is statistically significant [81].

As in other studies, we found that GPs, nurses and pharmacists were the most represented professionals in the IPC teams in the primary care setting [11, 85].

Several authors have shown that IPC would increases patient use and costs of care [86]. These indicators of the use and costs of the health care system are important from the perspective of the decision-maker, but they must be interpreted with caution. On the one hand, less use of to the health care system can testify to better individual health with lower morbidity (a reduction in the number of visits to the emergency room for example, or a reduction in the number of hospitalizations) [38, 66, 87]. On the other hand, these indicators can also attest to a positive effect with regard to issues of compliance and improvement of patient health: in many patients with chronic diseases monitored in primary care, the recommendations emphasize the prevention of serious events through the implementation of reinforced medical monitoring, resulting in an increase in the use of health care system by patients. Thus, the increase in the number of consultations with a health care professional may be appropriate during a period of antihypertensive treatment dosage adjustment to allow the achievement of the objectives [29].

### Strengths and limitations

This literature review has several strengths. First, it is the first to identify in a transversal way in which fields the effect of IPC has been analysed and in which fields this effect has been demonstrated. In 2020, Pascucci carried out a literature review and a meta-analysis on the subject, but without restricting its research equation to the primary care setting and only focusing on patients with chronic pathologies [88]. It concluded that IPC would improve the 3 following cardiovascular outcomes: BP, HbA1c levels, and the number of days of hospitalization in patients undergoing the IPC intervention. Second, our systematic review was based on the PRISMA quality guidelines; the research was carried out in 4 databases. Third, we assume that the focus on studies conducted in a primary care setting, involving primary care providers exclusively, might be a strength, while previous authors focused on the collaborations between primary and secondary care professionals.

This review also has limitations. The formalization of the search equation leading to the selection of studies required tedious work since there is no published search filter to search for articles addressing IPCs. It might be the main limitation of this review. Future work should consist of a specific and sensitive search filter developed by researchers in the field.

It was not possible to perform a bias analysis with the iCROMs [89] tool because the designs of the studies were too disparate. The quality of the studies was therefore assessed using the revised Downs and Black Checklist [18].

Effective studies are probably overrepresented compared to ineffective studies due to publication bias [90]. Similarly, many studies presented significant results but were based on post hoc or subgroup analyses.

The low level of evidence of clinical efficacy may be linked to the low level of internal validity of these studies, which are mainly pragmatic clinical trials [91, 92]. These trials are complex and therefore give positive results with greater difficulty.

In our review, the analysis of the differences between the control group and the multiprofessional intervention groups may have been biased by the fact that the primary care teams studied set up collaborative actions based on different concepts, e.g., the use of information technology, the training of health professionals or the therapeutic education of patients. Therefore, it is sometimes impossible to differentiate for these interventions whether the demonstrated effect is linked to the action implemented by a tool or solely by the IPC.

The concrete typology and level of collaboration among professionals differs greatly from one team to another, and this aspect was not the focus of our research. We therefore chose to describe IPC from the perspective of the most common definition and real life practice [8] without detailing the interactions involved in collaboration or its level. The type and level of interaction within the IPC teams depends on local contexts, dependent on national support for primary care and IPC [8, 9]. Reviews have already confirmed that, in this context and in the absence of new work on the subject, it is difficult to find a consensual definition of the typology of IPC [9, 11].

### Perspective

Primary care research within the framework of IPC must be able to invest in the field of primary prevention and screenings; currently, IPC is underrepresented in the fields of chronic diseases, cancers, vaccination, addictions, etc., as well as some fields mentioned above (locomotor, oncology) that have not benefited or in which little research with a high level of evidence exists. These areas have already been explored and described in the literature, but not with a comparative trial evaluation in the specific field of IPC in primary care. Our focus was quite narrow and doesn’t give a wide picture of the IPC in primary care.

The interventions offered to patients are often well described in the articles, but the description of the type of professionals and their levels of collaboration is often limited. For example, a patient will be able to benefit from an exchange regarding his pathology and treatment with a pharmacist, who will make recommendations that will be discussed with the doctor, but exchanges between health professionals are insufficiently described in terms of the methods, duration, and frequencies of interactions [43].

Further work should focus on the intensity of IPC and the elements needed to achieve effective IPC. The following organizational elements are necessary: health policies structuring primary care and funding the time needed to work together, networks with local governance, secure and shared IT systems for working together on patient records, trainings for primary care teams to learn how to work together in confidence without losing sight of the patient's objectives [93].

Some studies, such as that of Benedict in 2018, showed an improvement in the primary outcome measure in the short term (3 or 6 months) without long-term maintenance [22]. As multiprofessional primary care teams have to deal with an increasing number of patients with chronic pathologies, it would therefore be logical to hope for a long-term effect of these interventions: studies with longer period of follow-up would be needed in this context.

## Conclusion

Many studies have shown that IPC can improve the management of patients at cardiovascular risk. Other studies have investigated the effect of IPC in polypathological elderly patients and in patients with mental or physical disorders. For these pathologies, the number of studies remains limited, and the results are heterogeneous. Researchers should be encouraged to perform studies based on comparative designs: it would increase evidence on the positive effect and benefits of IPC on patient variables.

### Supplementary Information

**Additional file 1. **Revised Downs and Black checklist for assessment of methodological quality.

**Additional file 2. **Assessment of studies quality.

## Data Availability

The datasets used and/or analysed during the current study available from the corresponding author on reasonable request.

## Abbreviations

Adj: Adjusted
C: Control group
CI: Confidence interval
DBP: Diastolic Blood Pression
HSCL score: Hopkins Symptom Checklist score
HADS: Hospital Anxiety and Depression Scale
I: Intervention Group
IPC: Interprofessional Collaboration
LDL-c: Low Density Lipoprotein cholesterol
OR: Odds-Ratio
MPR: Medication Possession Ratio
MSDs: Musculoskeletal Disorders
NRd: Not Randomized
NS: Not Significant
P: Prospective
PACIC: Patient Assessment of Chronic Illness Care
PTSD: Post traumatic stress disorder
R: Retrospective
Rd: Randomised
Rd C: Randomised in Cluster
SBP: Systolic Blood Pression
SCL: Symptom Checklist-core depression
