# Supplementary material for: Interprofessional collaboration in primary care: what effect on patient health? A systematic literature review
Source: BMC Prim Care. 2023 Nov 29;24:253. doi: 10.1186/s12875-023-02189-0 (PMC10685527; doi:10.1186/s12875-023-02189-0)
Supplement: Supplementary file 2 — Additional file 2. Assessment of studies quality. [file 12875_2023_2189_MOESM2_ESM.docx]

*Additional file 2: Assessment of studies quality*

| AUTHOR YEAR | DESIGN | CATEGORY | *REPORTING (max= 11)* | *EXTERNAL VALIDITY (max= 3)* | *INTERNAL VALIDITY BIAS (max=7)* | *INTERNAL VALIDITY CONFOUNDING (max= 6)* | *POWER (max=1)* | **TOTAL (max =28)** | **QUALITY RATING** |
| --- | --- | --- | --- | --- | --- | --- | --- | --- | --- |
| Adler 2004 | P / R | Depression | 9 | 2 | 4 | 5 | 1 | **21** | High |
| Agarwal 2019 | P / R | Diabetes | 7 | 0 | 2 | 5 | 0 | **14** | Moderate |
| Aigner 2004 | R / R | Elderly people | 5 | 0 | 3 | 1 | 0 | **9** | Limited |
| Aragonès 2019 | P / R | Depression and chronic musculoskeletal pain | 7 | 2 | 5 | 5 | 1 | **20** | Moderate |
| Areán 2007 | P / R | Depression | 6 | 3 | 3 | 6 | 0 | **18** | Moderate |
| Barceló 2010 | P / R | Diabetes | 5 | 0 | 2 | 4 | 0 | **11** | Limited |
| Benedict 2018 | R / NR | Diabetes | 7 | 3 | 5 | 5 | 1 | **21** | High |
| Boult 2008 Leff 2009 | P / R | Elderly people and comorbidities | 8 | 2 | 4 | 4 | 0 | **18** | Moderate |
| Boult 2011 | P / R | General population | 8 | 2 | 3 | 4 | 0 | **17** | Moderate |
| Boyd 2010 | P / R | Elderly people and comorbidities | 8 | 2 | 3 | 4 | 0 | **17** | Moderate |
| Brown 2003 | P / NR | Elderly people | 6 | 0 | 3 | 1 | 0 | **10** | Limited |
| Burns 2015 | P / R | Elderly people (veterans) | 4 | 1 | 4 | 3 | 0 | **12** | Limited |
| Carter 2009 Chen 2013 | P / R cluster | Hypertension | 8 | 1 | 4 | 4 | 1 | **18** | Moderate |
| Carter 2015 | P / R cluster | Hypertension | 8 | 2 | 4 | 5 | 1 | **20** | Moderate |
| Carter 2018 | P / R cluster | Cardiovascular disease | 8 | 2 | 4 | 5 | 1 | **20** | Moderate |
| Chan 2011 | P / R | Anxiety and depression | 7 | 1 | 5 | 4 | 1 | **18** | Moderate |
| Chen 2010 | P / NR | Diabetes and hypertension | 6 | 0 | 4 | 1 | 0 | **11** | Limited |
| Choi 2015 | P / NR | Brain stroke | 5 | 2 | 3 | 1 | 0 | **11** | Limited |
| Chwastiak 2017 | R / NR | Diabetes | 6 | 2 | 3 | 2 | 0 | **13** | Limited |
| Dolovich 2019 | P / R | Elderly people | 8 | 1 | 3 | 5 | 1 | **18** | Moderate |
| Edwards 2012 | R/ NR | Diabetes | 8 | 1 | 4 | 3 | 0 | **16** | Moderate |
| ElGerges 2020 | P / R | Diabetes | 3 | 1 | 4 | 1 | 0 | **9** | Limited |
| Engel 2016 | P / R | Post traumatic stress disorder and depression | 9 | 2 | 4 | 5 | 1 | **21** | High |
| Finley 2002 | P / NR | Depression | 7 | 1 | 3 | 3 | 0 | **14** | Moderate |
| Finley 2003 | P / R | Depression | 7 | 2 | 3 | 5 | 1 | **18** | Moderate |
| Fokkens 2011 | P / NR | Diabetes | 7 | 0 | 4 | 2 | 0 | **13** | Limited |
| Furler 2017 | P / R | Diabetes | 9 | 2 | 4 | 5 | 1 | **21** | High |
| Heisler 2012 | P / R | Diabetes | 6 | 2 | 5 | 5 | 1 | **19** | Moderate |
| Hogg 2009 | P / R | Comorbidities | 7 | 0 | 4 | 5 | 1 | **17** | Moderate |
| Jameson 2010 | P / R | Diabetes | 10 | 2 | 5 | 6 | 1 | **24** | High |
| Jiao 2014 / Jiao 2015 | P / NR | Diabetes | 7 | 2 | 5 | 3 | 1 | **18** | Moderate |
| Kolk 2004 | P / R | Medically unexplained symptoms | 5 | 1 | 4 | 4 | 1 | **15** | Moderate |
| Lenaghan 2007 | P / R | Elderly people | 7 | 2 | 4 | 3 | 1 | **17** | Moderate |
| Lin, 2014 | P / R | Depression / diabetes/ cordépression, coronaropathy | 6 | 3 | 3 | 5 | 0 | **17** | Moderate |
| Manns 2012 | R / NR | Diabetes | 8 | 1 | 5 | 1 | 0 | **15** | Moderate |
| Marklund 1999 | P / NR | Muskloskeletal disorders | 6 | 0 | 5 | 0 | 0 | **11** | Limited |
| Matzke 2018 | P / NR | Comorbidities | 9 | 0 | 4 | 1 | 0 | **14** | Moderate |
| McAdam-Marx 2015 | R/ NR | Diabetes | 9 | 1 | 4 | 2 | 0 | **16** | Moderate |
| Melis 2008 | P / R pseudocluster | Elderly people | 9 | 1 | 5 | 3 | 1 | **19** | Moderate |
| Morgan 2013 | P / R | Depression by diabetes or cardiopathy patients | 9 | 1 | 5 | 4 | 1 | **20** | Moderate |
| Mousquès 2010 | P / NR | Diabetes | 5 | 2 | 4 | 1 | 0 | **12** | Limited |
| Mundt 2015 | R/ NR | Cardiovascular disease | 6 | 3 | 3 | 1 | 0 | **13** | Limited |
| Pape 2011 | P / R | Diabetes | 8 | 3 | 4 | 5 | 1 | **21** | High |
| Petersen 2014 | P / R | Depression | 8 | 2 | 4 | 4 | 0 | **18** | Moderate |
| Riverin 2017 | R/ NR | Elderly people and comorbidities | 8 | 3 | 4 | 3 | 0 | **18** | Moderate |
| Rollman 2005 | P / R | Anxiety | 8 | 1 | 5 | 5 | 1 | **20** | Moderate |
| Schaefert 2013 | P / R Cluster | Medically unexplained symptoms | 8 | 1 | 5 | 5 | 1 | **20** | Moderate |
| Sellors 2003 | P / R | Elderly people | 8 | 1 | 4 | 4 | 1 | **18** | Moderate |
| Sherbourne, 2001 | P / R | Depression | 8 | 0 | 4 | 4 | 0 | **16** | Moderate |
| Simon 1998 | P / R | Depression | 6 | 1 | 3 | 4 | 0 | **14** | Moderate |
| Simpson 2011 Omran 2015 | P / R | Diabetes | 9 | 1 | 5 | 5 | 1 | **21** | High |
| Smith 2004 | P / R | Diabetes | 7 | 1 | 4 | 5 | 1 | **18** | Moderate |
| Smith 2016 | P / R | Hypertension | 8 | 2 | 4 | 4 | 1 | **19** | Moderate |
| Sommers 2000 | P / R | Elderly people | 7 | 1 | 4 | 4 | 1 | **17** | Moderate |
| Tahaineh 2011 | P / R | Dyslipidemia | 8 | 0 | 5 | 4 | 0 | **17** | Moderate |
| Taplin 1998 | P / NR | General population | 6 | 3 | 5 | 2 | 0 | **16** | Moderate |
| Tobari 2010 | P / R | Hypertension | 8 | 2 | 5 | 5 | 1 | **21** | High |
| Van Lieshout 2018 | P / R | Elderly people | 9 | 3 | 3 | 4 | 1 | **20** | Moderate |
| Vitale 2020 | P / R | Diabetes | 6 | 3 | 3 | 4 | 1 | **17** | Moderate |
| Weber 2010 | P / R cluster | Hypertension | 7 | 2 | 4 | 4 | 1 | **18** | Moderate |
| Wolff 2010 | P / R | Comorbidities | 7 | 1 | 5 | 4 | 1 | **18** | Moderate |

P = Prospective R = Retrospective Rd= randomised, Rd PC = Pseudo cluster randomised NRd= nonrandomised MSDs=Musculoskeletal disorders, PTSD= Post traumatic stress disorder
